# A Monocular Depth Estimation Method for Autonomous Driving Vehicles Based on Gaussian Neural Radiance Fields

**DOI:** 10.3390/s26030896

**Published:** 2026-01-29

**Authors:** Ziqin Nie, Zhouxing Zhao, Jieying Pan, Yilong Ren, Haiyang Yu, Liang Xu

**Affiliations:** 1 School of Transportation Science and Engineering, Beihang University, Beijing 102206, China; zqnie@buaa.edu.cn (Z.N.); jypan@buaa.edu.cn (J.P.); yilongren@buaa.edu.cn (Y.R.); hyyu@buaa.edu.cn (H.Y.); 2 Hangzhou Innovation Institute, Beihang University, Hangzhou 310023, China; 3 The State Key Lab of Intelligent Transportation System, Beijing 100191, China; 4 Zhejiang Communications Services Co., Ltd., Hangzhou 310051, China; zhaozhouxing.zj@chinaccs.cn

**Keywords:** autonomous driving, monocular depth estimation, Neural Radiance Fields, Gaussian-Probability sampling, adaptive channel attention

## Abstract

Monocular depth estimation is one of the key tasks in autonomous driving, which derives depth information of the scene from a single image. And it is a fundamental component for vehicle decision-making and perception. However, approaches currently face challenges such as visual artifacts, scale ambiguity and occlusion handling. These limitations lead to suboptimal performance in complex environments, reducing model efficiency and generalization and hindering their broader use in autonomous driving and other applications. To solve these challenges, this paper introduces a Neural Radiance Field (NeRF)-based monocular depth estimation method for autonomous driving. It introduces a Gaussian probability-based ray sampling strategy to effectively solve the problem of massive sampling points in large complex scenes and reduce computational costs. To improve generalization, a lightweight spherical network incorporating a fine-grained adaptive channel attention mechanism is designed to capture detailed pixel-level features. These features are subsequently mapped to 3D spatial sampling locations, resulting in diverse and expressive point representations for improving the generalizability of the NeRF model. Our approach exhibits remarkable performance on the KITTI benchmark, surpassing traditional methods in depth estimation tasks. This work contributes significant technical advancements for practical monocular depth estimation in autonomous driving applications.

## 1. Introduction

Environmental perception serves as a fundamental pillar of autonomous driving systems, as the reliability of tasks such as motion planning and decision-making relies heavily on accurate, robust and real-time understanding of surrounding scenes [1,2,3,4]. Unlike common LiDAR-based perception methods, which provide accurate depth measurements through active sensing [5,6], monocular depth estimation infers scene geometry from visual cues and thus offers a low-cost and flexible complementary perception solution. It plays a particularly important role because it enables the extraction of 3D structural information from easily accessible monocular images, providing a practical and scalable way to support scene understanding in autonomous driving [7,8,9]. Despite their advantages, existing monocular depth estimation methods still exhibit several limitations that hinder their reliability in real-world autonomous driving scenarios. Firstly, the inherent scale ambiguity of monocular imagery makes it difficult to determine absolute depth, often causing noticeable degradation in estimation accuracy particularly for distant objects. In addition, the highly diverse appearance and layout of urban traffic scenes impose further challenges, causing noticeable fluctuations in the quality of predicted depth maps. These limitations collectively restrict the robustness of monocular depth perception and highlight the need for more adaptive and generalizable estimation frameworks.

Neural Radiance Fields (NeRF) [10] have achieved remarkable breakthroughs in the field of 3D vision. By implicitly modeling scene radiance fields with multilayer perceptrons, NeRF demonstrates strong capability in reconstructing complex geometric structures. This powerful 3D representation offers a promising direction for addressing long-standing issues in monocular depth estimation, such as incomplete structural recovery and limited geometric consistency. Nevertheless, despite its potential, existing NeRF-based depth estimation approaches still encounter two major challenges in practical deployment. Firstly, maintaining depth accuracy for long-range rays requires dense sampling in conventional NeRF, resulting in substantial computational and memory overhead in hundred-meter driving scenarios, which limits deployment on resource-constrained onboard platforms. Secondly, existing methods are typically trained on single scenes and generalize poorly across environments. Models optimized on indoor data suffer significant degradation when applied to urban road scenes, failing to achieve the cross-scene demand of autonomous driving. Thus, improvements in sampling strategies and network designs are needed to enhance efficiency and generalization.

Despite the promising progress of existing NeRF-based monocular depth estimation methods, they still suffer from high computational and memory costs caused by dense ray sampling in long-range driving scenes and exhibit limited generalization in complex urban environments. These limitations restrict their practical deployment in real-world autonomous driving systems. To address these challenges, we introduce a NeRF-based depth estimation framework that integrates Gaussian-probability ray sampling with a spherical network enhanced by fine-grained adaptive channel attention. The proposed method incorporates two key innovations. First, to improve sampling efficiency in long-range environments, a one-dimensional Gaussian mixture is employed to approximate the density distribution along each ray, enabling sampling points to be concentrated near likely surface locations. This strategy substantially reduces the number of samples required for hundred-meter scenes, thereby lowering computational and memory costs. Second, to enhance scene generalization, a lightweight spherical U-Net is designed to expand the effective field of view and extract more expressive pixel-level features. These features are then projected onto 3D sampling positions to provide rich geometric cues for NeRF optimization. Extensive experiments on the KITTI benchmark demonstrate that the proposed method achieves accurate long-distance depth estimation and delivers more reliable reconstruction in occluded regions than existing NeRF-based approaches. These results confirm the effectiveness of the proposed framework in large-scale and structure-complex environments, offering practical value for the deployment of visual perception systems in autonomous driving and contributing to the development of cost-efficient, high-performance sensing solutions.

## 2. Related Work

### 2.1. Monocular Depth Estimation via Supervision-Based Techniques

Neural network driven approaches to monocular depth estimation recover depth information directly from a single RGB input. Among these methods, supervision-based monocular depth estimation uses ground-truth depth maps to train the network, penalizing the discrepancy between the predicted depth and the ground truth. There are many relevant studies in this field. Li et al. proposed a network structure based on VGG-16 [11] for depth estimation [12], which estimates depth through a depth regression part and a gradient fusion module. In addition to using ordinary convolutions for estimation, some methods also use complex convolution-based approaches to learn depth information corresponding to each pixel in the image, including methods using VGG modules [13], ResNet [14], and DenseNet [15]. The above methods all estimate actual depth. Although absolute depth estimation achieves good results in accuracy, its models still have defects in robustness, i.e., the models are easily affected in practical applications. Therefore, Zoran et al. proposed a method for estimating relative depth of images [16], which inputs the relationship between corresponding points in two images into the network and optimizes it with numerical values to obtain dense depth information. The robustness of the model is significantly enhanced by estimating relative depth information. Furthermore, Chen et al. used a multi-scale network and supervised relative depth through loss functions [17]. The entire training process recovers depth information without constraints. Finally, the inferred depth map is evaluated to have a root mean square error (RMSE) of 1.10, which is comparable to the performance of absolute depth estimation models on this evaluation index [18]. Lee et al. optimized and reorganized the depth confidence estimated at different scales through CNN based on this, and the final depth map is the optimal result of integrating depth maps at different scales [19].

Subsequently, some researchers introduced the method of conditional random field (CRF) into monocular depth estimation. The so-called CRF is a conditional probability distribution model for input sequences [20]. Xu et al. [21] combined this method with an attention mechanism enabling the model to automatically learn more robust multi-scale features. Ricci et al. achieved the integration of multi-scale feature information by cascading multiple CRFs [22]. In addition to the method of cascading CRFs, many methods combine CNN with continuous CRF [23], hierarchical CRF [24] and FC-CRF [25] to estimate depth information.

While the estimation accuracy has been notably improved by supervised learning techniques, their specific implementation requires datasets annotated with real depth information and the acquisition of such datasets often requires high-cost LiDAR, which runs counter to the original intention of using deep learning for depth estimation. Therefore, researchers introduce a self-supervised depth estimation method that do not require training with real depth information.

### 2.2. Monocular Depth Estimation via Self-Supervised Learning

Self-supervised monocular depth estimation is typically trained using either stereo pairs with known baselines or consecutive frames from monocular videos. Although only a single image is required at inference time, these methods learn depth by exploiting multi-view relationships present during training. Most existing approaches derive supervisory signals by enforcing geometric or appearance consistency across views.

A wide range of self-supervised monocular depth estimation methods have been developed around the idea of using image reconstruction as supervisory signals. Early work introduced view-synthesis-based training, in which one view is warped to another and the reconstruction error is minimized to infer depth [26]. This paradigm was later expanded with stronger geometric constraints. For example, left–right consistency strategies [27] and selective reconstruction layers [28] were proposed to stabilize stereo-based supervision, while Siamese feature extractors were explored to enhance correspondence learning across paired images [29]. Another line of research focused on enriching feature representations to improve pixel-level matching. Global-to-local feature extraction networks [30], depth-cue-guided matching mechanisms [31] and unsupervised pretrained filtering modules [32] were introduced to obtain more reliable structural correspondences across views. More advanced approaches incorporated spatial pyramid pooling modules [33]. to aggregate multi-scale context and build more expressive cost volumes, which are subsequently refined through stacked hourglass-based 3D convolution layers with intermediate supervision [34].

Since self-supervised methods trained with binocular image pairs are susceptible to mapping relationships between images, some researchers have proposed using monocular image sequences for training. Monocular sequences enable reconstruction of image projection mappings during model training, which positively impacts monocular depth estimation [35]. A common model structure consists of two networks: a pose network for visual odometry estimation [36] and a depth estimation network for depth information. Zhou et al. were the first to propose joint training of two networks [37], which contains a camera pose estimation network and a depth estimation network. Also, they further introduced photometric consistency and view-reconstruction losses to regularize training [38], enforcing that corresponding pixels across views maintain similar appearance. These geometry-driven constraints became foundational in subsequent self-supervised depth estimation works. Nevertheless, due to the absence of ground-truth depth supervision, self-supervised approaches generally remain less accurate than supervised methods.

### 2.3. NeRF-Based Monocular Depth Estimation Methods

Recent progress in neural implicit representations has pushed NeRF to the forefront of 3D vision due to its ability to model complex scene geometry and appearance with high fidelity [39]. These strengths naturally motivate its adoption in monocular depth estimation, where depth must be inferred from limited visual cues. However, classical NeRF formulations were originally designed for controlled settings that provide dense multi-view observations and accurate camera poses. In such pipelines, the radiance field is optimized through computationally expensive volumetric rendering and MLP-based implicit representations, making them ill-suited for monocular scenarios that lack explicit metric depth cues. As a result, NeRF-based depth inference remains susceptible to scale ambiguity and unstable reconstruction in occluded or texture-poor regions. These limitations have driven extensive efforts to redesign NeRF representations and training strategies toward more efficient and adaptable monocular depth estimation.

To improve computational efficiency, recent efforts to accelerate NeRF have introduced highly efficient representations such as hash-based feature encoding and sparse voxel structures, as exemplified by Instant-NGP [40]. These designs substantially reduce the computational burden associated with querying radiance fields, making real-time or near real-time NeRF inference increasingly feasible for monocular depth estimation pipelines. In parallel, several studies have explored integrating monocular depth cues into NeRF optimization to alleviate its dependence on dense multi-view supervision. A representative work, NoPe-NeRF [41], incorporates depth maps predicted by monocular networks into the joint optimization of Pose-NeRF. By adjusting scale and offset to enforce multi-view geometric consistency, this approach enhances both radiance field reconstruction and pose estimation, particularly under challenging camera trajectories. Such strategies effectively mitigate NeRF’s sensitivity to inaccurate camera poses and extend its applicability in monocular settings.

Considering the inherent uncertainty and ambiguity present in monocular depth estimation outputs, the SCADE method [42] models the probability distribution of depth estimation, introduces spatial cutting loss. Also, it integrates depth estimation data from multiple perspectives, which realizes higher-fidelity novel view synthesis from sparse views and compensates for the insufficient constraints in the few-view areas. In terms of scene adaptability, some studies focus on specific scenes such as indoor scenes combining monocular dense SLAM with NeRF. For instance, NeRF-SLAM [43] estimates camera poses, dense depth maps and their uncertainties through monocular dense SLAM. The method uses them as supervision signals to train NeRF scene representations and achieves real-time and accurate scene reconstruction.

Despite significant progress in NeRF-based monocular depth estimation, several challenges persist. The model’s capacity to adapt to complex and dynamic environments is constrained, especially when dealing with fast-moving objects and fluctuating lighting conditions. What’s more, the model’s generalization capability is insufficient, resulting in significant performance degradation when applied to different scenes and datasets. Our work addresses the key issues in autonomous driving monocular depth estimation by introducing a NeRF-based framework that integrates a Gaussian probability sampling strategy and an adaptive channel attention mechanism. By refining the ray sampling distribution and enhancing the feature fusion network, our method enables efficient depth estimation and robust scene understanding in large-scale autonomous driving scenarios.

## 3. Methodology

This paper infers the geometric shape of scenes from monocular RGB images and performs self-supervised training using an image conditional NeRF model. Depth information is deduced from the NeRF radiance volume and optimized via a reprojection loss function. The inputs to the NeRF model include extracted feature vectors, encoded sampling point position coordinates and viewing directions.

**Figure 1:**
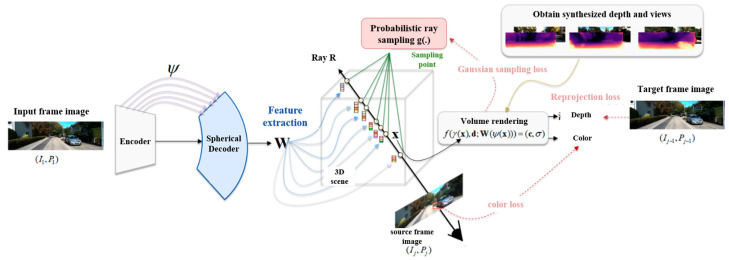
Architecture of NeRF-based monocular depth estimation method for autonomous driving vehicles.

To estimate depths in large complex scenes and endow the model with generalization ability, this method proposes a spherical network based on an adaptive fine-grained channel attention mechanism to extract image features and generate universal and sampling point representations. Additionally, a Gaussian probability-based ray sampling method is introduced to sample points close to surfaces. It reduces the number of sampling points in large autonomous driving scenes. The training data of the model contains S sequences, each includes m RGB images and corresponding pose information, specifically denoted as ${\left\{\left(I_{1}^{i},P_{1}^{i}),\ldots ,(I_{m}^{i},P_{m}^{i}\right)\right\}}_{i=1}^{S}$. This method estimates the neural representation conditioned on each first frame ${\left\{I_{1}^{i}\right\}}_{i=1}^{S}$ and learns a radiance field shared among sequences. The specific implementation is illustrated in Figure 1.

### 3.1. Spherical Network Based on Channel Attention Mechanism

NeRF-based monocular depth estimation methods generally require scene-specific retraining and exhibit limited generalization to unseen environments. A key factor underlying this limitation lies in their reliance on conventional encoder–decoder architectures for 2D feature extraction. Standard U-Net structures confine learned features to the camera’s field of view (FOV), preventing NeRF from inferring colors and depths beyond visible regions. Moreover, repeated downsampling–upsampling operations tend to produce ambiguous or degraded features, which weakens the effectiveness of projected 3D point representations.

To overcome these shortcomings, this paper introduces a spherical U-Net enhanced with a fine-grained adaptive channel attention mechanism. Through spherical projection, 2D features are mapped onto a wider angular domain, enabling the network to incorporate contextual information beyond the original FOV and construct richer 3D point descriptors. Meanwhile, the adaptive channel attention module dynamically fuses global contextual cues with local geometric details, delivering more accurate feature weighting and substantially enhancing the discriminative ability of extracted features.

Building on this design, the decoder is further restructured to operate on a spherical surface, reducing geometric distortion compared with planar projection and effectively expanding the usable FOV to approximately 120°. This extension allows the network to recover depth and color information from regions that would otherwise lie outside the image boundary. To mitigate the feature degradation commonly introduced during upsampling, the proposed fine-grained channel attention (FCA) module explicitly models both global and local dependencies. Unlike SE, which focuses mainly on global statistics through fully connected layers, the proposed FCA module integrates both global and local cues, enabling more accurate channel weighting and improved generalization.

**Figure 2:**
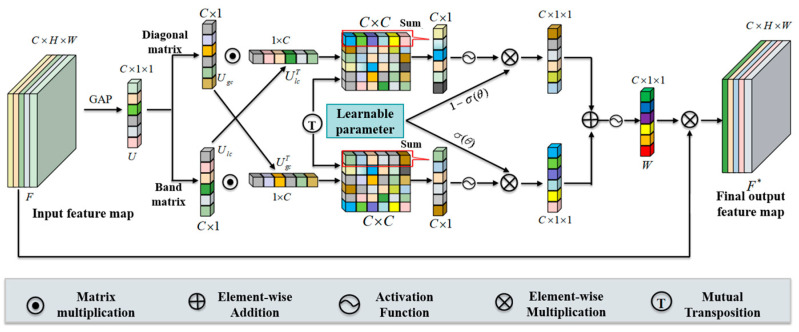
Architecture of the adaptive fine-grained channel attention module.

In the spherical U-Net, the adaptive fine-grained channel attention module is primarily applied in the decoder, where feature refinement is essential. For view-synthesis tasks, combining global context with local channel cues improves the suppression of blur and enhances reconstruction fidelity. As illustrated in Figure 2, firstly, to summarize channel-level responses from the feature maps, this method converts the feature map F containing global spatial information into a channel descriptor U through global average pooling. Given the feature map $F\in R^{C\times H\times W}$, where C, H and W denote the number of channels, height and width, respectively. The channel descriptor $U\in R^{C}$ is generated via GAP. The n-th channel element of U is expressed by Equation (1):

(1) $$U_{n}=GAP\left(F_{n}\right)=\frac{1}{H\times W}\sum_{i=1}^{H}\sum_{j=1}^{W}F_{n\left(i,j\right)}$$

Here, $F_{n}(i,j)$ denotes the activation at spatial location (i,j) in the n-*th* channel of the feature map, while GAP(x) refers to the global average pooling function. This function compresses the feature map F from C×H×W to C×1×1. To obtain local channel information while maintaining fewer model parameters, a matrix B is used for local channel interaction with the setting $B=[b_{1},b_{2},b_{3},\ldots ,b_{k}]$. This leads to Equation (2):

(2) $$U_{lc}=\sum_{i=1}^{k}U\cdot b_{i}\;\;$$

where U denotes the channel descriptor, $U_{lc}$ represents the local information, and k signifies the number of adjacent channels. In this experiment, a one-dimensional convolution (conv1D) is employed to implement this module. To obtain global channel information and enhance the capability of representing global context, a diagonal matrix D is utilized to capture dependencies among all channels as global information, with $D=[d_{1},d_{2},d_{3},\ldots ,d_{c}]$. This yields Equation (3):

(3) $$U_{gc}=\sum_{i=1}^{c}U\cdot d_{i}\;\;$$

where $U_{gc}$ denotes the global information and c represents the number of channels, a two-dimensional convolution is used to implement this module. To enable meaningful integration of global and local cues, the global features derived from the diagonal matrix are fused with the local features produced by the weight matrix. Finally, cross-correlation operations are employed to capture the correlations between them at various granularities, with the specific form shown in Equation (4):

(4) $$M=U_{gc}\cdot U_{lc}^{T}\;\;$$

Here, M represents the correlation matrix. To balance accurate feature weighting with computational efficiency, an adaptive fusion strategy is introduced. This mechanism constructs global and local weight vectors by extracting row- and column-level statistics from M and its transpose, respectively, and subsequently merges them using learnable fusion coefficients, as formalized in Equations (5)–(7):

(5) $$U_{gc}^{w}=\sum_{j}^{c}M_{i,j}\;(i\in 1,2,3,\ldots ,c)$$

(6) $$U_{lc}^{w}={\sum_{j}^{c}\left(U_{lc}\cdot U_{gc}^{T}\right)}_{i,j}=\sum_{j}^{c}M_{i,j}^{T}\;(i\in 1,2,3,..,c)\;\;$$

(7) $$W=\sigma (\sigma (\theta )\times \sigma (U_{gc}^{w})+(1-\sigma (\theta ))\times \sigma (U_{lc}^{w}))\;\;$$

where $U_{gc}^{w}$ and $U_{lc}^{w}$ denote the fused global and local channel weights, respectively, c is the number of channels, and θ is a learnable parameter. This design eliminates unnecessary cross-correlation computations between global and local representations while strengthening their mutual interaction. As a result, the mechanism selectively amplifies informative channels and suppresses irrelevant ones, yielding more accurate weight assignments for deblurring-related features. The resulting weights are then applied to the input feature map, as indicated in Equation (8) where F represents the feature map, and $F^{*}$ denotes the final output feature map:

(8) $$F^{*}=W⊗F\;\;$$

**Figure 3:**
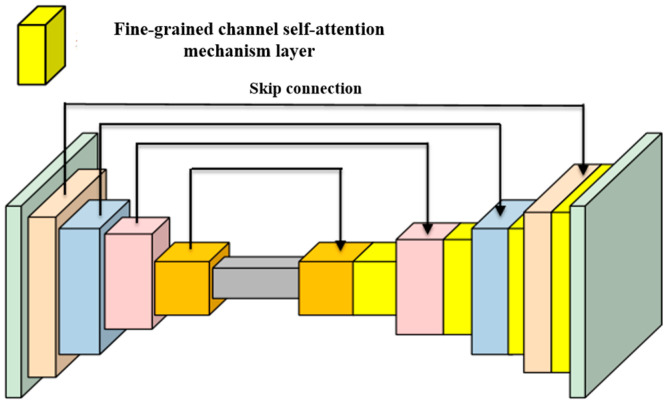
Spherical network architecture based on adaptive fine-grained channel attention mechanism.

At the network bottleneck, features are transformed onto a spherical surface using ψ(⋅) before entering the spherical decoder. To accommodate the expanded feature domain, the decoder applies lightweight dilated convolutions, enabling a larger receptive field at low cost. Following the U-Net design principle, multi-scale skip connections are employed to maintain effective gradient flow, requiring only feature remapping through ψ(⋅). The encoder leverages a pretrained EfficientNet-B7 for 2D feature extraction, while the spherical decoder comprises five stages that upsample resolution and progressively reduce channel depth. Each layer incorporates an adaptive fine-grained channel self-attention module. To compensate for the large blank areas caused by the expanded field of view, three ResNet blocks with dilation rates of 1, 2 and 3 are embedded in each layer to enhance the receptive field. Additionally, skip connections are applied between the encoder and decoder at corresponding scales. The specific network architecture is illustrated in Figure 3.

In the experiment, each 2D pixel $[x,y{]}^{T}$ is converted into its normalized spherical coordinates [θ,ϕ]. Given that the vector $[\nabla_{x},\nabla_{y},1{]}^{T}∼K^{-1}[x,y,1{]}^{T}$ represents the viewing ray originating from the camera center and passing through that pixel, the corresponding spherical projection can be formulated as in Equation (9):

(9) $$\psi \left(\begin{array}{c} x\\ y \end{array}\right)=\left(\begin{array}{c} \theta \\ \phi \end{array}\right)=\left(\begin{array}{c} \pi -\arctan(\nabla_{x}^{-1})\\ \arccos(\frac{-\nabla_{y}}{r}) \end{array}\right)\;\;$$

where $r=\sqrt{\nabla_{x}^{2}+\nabla_{y}^{2}+1}$. When input into the decoder, [θ,ϕ] are uniformly discretized, and features are stored in a tensor covering an arbitrarily large FOV. Through the above modules, given an input image, new depth views can be uniformly synthesized at different angles along the imaginary straight path.

### 3.2. Feature-Informed NeRF Color Prediction

In its standard formulation, NeRF models a continuous volumetric radiance field f(⋅)=(σ,c) that maps a 3D location $x\in R^{3}$ and viewing direction $d\in R^{3}$ to two quantities: the volume density σ and RGB color c. Building upon PixelNeRF, this method learns a generalizable cross-sequence radiance field and introduces novel sampling designs for efficiently synthesizing new depth views.

The basic architecture is shown in Figure 1. Using the first frame as input $I_{1}$ in Sequence 1, a spherical U-Net with adaptive fine-grained attention extracts a feature volume $W=E(I_{1})$. A source future frame $I_{j},2\leq j\leq m$ is randomly selected, from which l pixels are sampled. Using known source poses and camera intrinsics, N points are efficiently sampled along rays passing through these pixels. Each sampled point x is projected onto a sphere via ψ(⋅), allowing corresponding input image feature vectors W(ψ(x)) to be retrieved by bilinear interpolation. These features W(ψ(x)), combined with viewing direction d and positional encoding γ(x), are fed into NeRF’s multi-layer perceptron f(⋅) to predict point density σ and RGB color c. in the input frame coordinates, as formalized in Equation (10):

(10) f(γ(x),d;W(ψ(x)))=(c,σ)  

Following the NeRF formulation, the color $\hat{C}(r)$ is computed by numerically aggregating the radiance samples along ray r. Its generalized expression is provided in Equation (11):

(11) $$\hat{C}(r)=\sum_{i}^{N}w_{i}c_{i}\;\;$$

where $w_{i}=T_{i}(1-\exp(-\sigma_{i}\delta_{i}))$, with $T_{i}$ denoting the cumulative transmittance and $\delta_{i}$ the distance to the previous adjacent point. Unlike traditional self-supervised methods, this approach disentangles depth from the radiance volume and defines the estimated depth as the distance from sampling points to the object surface.

### 3.3. Monocular Depth Estimation Method via Neural Radiance Field

Similarly to the color prediction method in the previous subsection, the depth $\hat{D}\left(r\right)$ estimated by NeRF is defined in the form shown in Equation (12):

(12) $$\hat{D}\left(r\right)=\sum_{i}^{N}w_{i}d_{i}\;\;$$

Here, $d_{i}$ denotes the distance between the i-*th* sampled point and its corresponding sampling location. To enable depth optimization without ground-truth annotations, the method follows conventional self-supervised monocular depth estimation paradigms by employing a photometric reprojection loss between the warped source image $I_{j}$ and its preceding frame $I_{j-1}$ (i.e., the target frame). Meanwhile, continuous frames are selected to ensure maximum overlap. For the sparse depth estimation ${\hat{D}}_{j}$, the photometric reprojection loss $L_{\mathrm{reproj}}$ is expressed as Equation (13):

(13) $$L_{\mathrm{reproj}}=\frac{1}{l}{\sum_{i=1}^{l}\left\|I_{j}(i)-I_{j-1}\left(\mathrm{proj}({\hat{D}}_{j}(i))\right)\right\|}_{1}\;\;$$

where proj(⋅) denotes the projection of 2D coordinate i onto image $I_{j-1}$, using the camera’s intrinsic parameters and poses. Although ${\hat{D}}_{j}$ obtained in this method is sparse (since it is estimated only for certain rays), the randomness of these rays provides statistically dense supervision. To account for moving objects in autonomous driving scenarios, this method also applies a pixel-wise auto-masking strategy during depth estimation.

To reduce the number of sampling points for NeRF in large-scale scenarios such as autonomous driving, this chapter proposes a Gaussian probability-based sampling strategy that incorporates the depth prior information predicted above. This strategy models the density distribution along each ray using a one-dimensional Gaussian mixture guided by the sampled points. Because higher mixture responses typically indicate proximity to object surfaces, the method can concentrate sampling in more informative regions, thereby reducing the number of required samples.

### 3.4. Gaussian Probability-Based Ray Sampling Method

To mitigate this problem, a Gaussian-based probabilistic sampling strategy is adopted, approximating the ray’s density profile with a 1D Gaussian mixture estimated from sampled points. As peaks in the mixture align with likely surface locations, the method can focus sampling accordingly and greatly reduce the number of required samples using just 64 points for a 100 m ray.

**Figure 4:**
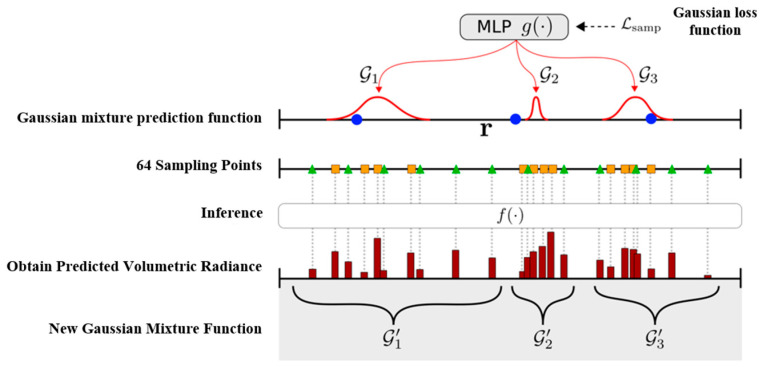
Probabilistic ray sampling steps.

As shown in Figure 4, for a given ray r, first, k points (blue dots in the figure) are uniformly sampled at the near and far ends. Taking the blue sampling points and their features as inputs, an MLP network g(⋅) is used to predict k 1D Gaussian mixtures $\left\{G_{1},\ldots ,G_{k}\right\}$. Then, m points (square points in the figure) are sampled from each Gaussian distribution and 32 points (triangular points in the figure) are uniformly sampled along the ray with a total of N=k×m+32 points sampled.

Where the additional uniform point sampling enforces calculations on the radiance volume to prevent g(⋅) from getting into local minima. All sampled points are then fed into f(⋅) in Equation (10) for NeRF volume rendering of color $\hat{C}(r)$ and depth $\hat{D}(r)$. The inferred densities $\left\{\sigma_{1},\ldots ,\sigma_{N}\right\}$ during rendering serve as cues for 3D surface positions, from which new Gaussian mixtures can be obtained—but this requires solving a point-Gaussian assignment problem. Thus, this chapter proposes a probabilistic self-organizing map (PSOM) method to address this issue, which is shown in the Algorithm 1. In this framework, sampling points are associated with individual Gaussian components according to the probability that each point is generated by that component, while the structure of the mixture is strictly maintained. For a Gaussian $G_{I}$ and its assigned point set $\chi_{i}$, the updated Gaussian $G_{i}^{’}$ is the mean of all points $j\in \chi_{i}$, weighted by the conditional probability $p(\frac{j}{G_{i}})$ where $\alpha_{j}$ is the occupancy of j. In specific experiments, α from the original NeRF formulation is used as it serves as a sufficiently good occupancy estimator, i.e., $\alpha_{j}=1-\exp(-\sigma_{j}\delta_{j})$, where $\delta_{j}$ is the distance to the previous point.
**Algorithm 1:** PSOM-based Point–Gaussian AssignmentInput: Sampling points {$x_{j}$}, Gaussian components {$G_{i}$}Output: Updated Gaussian components {$G_{i’}$} 1: Render sampling points with NeRF to obtain densities $\sigma_{j}$2: Compute occupancy $\alpha_{j}$ = 1 − exp($-\sigma_{j}$ $\cdot \delta_{j}$)3: for each sampling point $x_{j}$ do4:    for each Gaussian component $G_{i}$ do5:       Compute conditional probability p(j|$G_{i}$)6:    end for7: end for8: for each Gaussian component $G_{i}$ do9:    Update $G_{i}^{\prime }$ using the weighted mean of assigned points with p(j|$G_{i}$) and $\alpha_{j}$10: end for11: Compute Gaussian consistency loss:12:    L_gauss = (1/k) $\sum_{i}KL(G_{i}|G_{i}^{\prime })$13: Compute surface consistency loss L_surface14: L_samp = L_gauss + L_surface15: Update Gaussian predictor g(·) by minimizing L_samp

Finally, the Gaussian predictor g(⋅) is subsequently updated by computing the average KL divergence between the current and revised Gaussian components, as expressed in Equation (14):

(14) $$L_{\mathrm{guass}}=\frac{1}{k}\sum_{i}^{k}\mathrm{KL}(G_{i}∥G_{i}^{’})\;\;$$

To further enforce a Gaussian on visible surfaces, this method also minimizes the distance between the depth and the nearest Gaussian. The total loss is: $L_{\mathrm{samp}}=L_{\mathrm{gauss}}+L_{\mathrm{surface}}$. In experiments, k=4 Gaussian functions are used, with each Gaussian sampling m=8 points, and 32 points are uniformly sampled, such that each ray only requires =64 sampling points.

## 4. Experiments

### 4.1. Experimental Datasets

This paper utilizes the KITTI dataset, which is widely used in the fields of autonomous driving, robotic navigation and computer vision. The dataset is designed to provide standardized data support for various visual tasks, particularly real-time navigation and 3D environmental modeling. The scenes in the dataset primarily cover urban areas, rural regions and highways. This study employs the visual odometry subset of the dataset, which contains 22 image sequences with a total length of 39.2 km. Specifically, the method uses the image_2 monocular camera images and the corresponding pose information for experimentation. To evaluate the model’s performance, all sequences except Sequence 08 are used to train, with Sequence 08 reserved for validation.

The SemanticKITTI dataset is a large-scale benchmark designed for object detection and semantic segmentation in autonomous driving. It was created by the Computer Vision Group at the University of Trier in Germany as an extension and enhancement of the original KITTI dataset. Focused on providing dense semantic supervision for LiDAR point clouds, SemanticKITTI facilitates advanced research in LiDAR-based semantic segmentation and object detection. The dataset includes 22 sequences of LiDAR point cloud data, each with point-wise annotations across 28 categories such as roads, buildings, vehicles, pedestrians. These annotations not only distinguish between stationary and moving objects but also provide rich contextual cues, enabling autonomous systems to better perceive and interpret their surroundings. In this study, the depth information from the dataset is primarily used to evaluate the experimental results.

### 4.2. Experimental Metrics and Settings

In this paper, the depth is limited to 80 m and the common metrics are calculated: the average absolute relative error (Abs Rel: $\frac{1}{\left|T\right|}\sum_{y\in T}\left|y-y^{*}\right|/y^{*}$), the squared absolute relative error ($\mathrm{Sq}\;\mathrm{Rel}:\frac{1}{\left|T\right|}{\sum_{y\in T}\left\|y-y^{*}\right\|}^{2}/y^{*}$), the root mean square error (RMSE: $\sqrt{\frac{1}{\left|T\right|}{\sum_{y\in T}\left\|y-y^{*}\right\|}^{2}}$), the average log_10_ error (RMSE log: $\sqrt{\frac{1}{\left|T\right|}{\sum_{y\in T}\left\|\log y-\log y^{*}\right\|}^{2}}$) and the threshold accuracy (δ$:\max(\frac{y}{y^{*},\frac{y^{*}}{y})}<\tau (\tau =1.25/1.25^{2}/1.25^{3})$), where y and $y^{*}$ denote the predicted depth value and the ground-truth depth value, respectively. And T represents all pixels in the depth map.

We use the following losses for end-to-end training and the total loss function is shown in Equation (15), where $L_{reproj}$ is the photometric loss, and $L_{rgb}$ is the standard L2 photometric reconstruction loss used in NeRF:

(15) $$L_{total}=L_{reproj}+L_{rgb}+L_{samp}\;\;$$

Experiments were executed on Ubuntu 18.04 with four RTX 3090 Ti GPUs (24 GB, CUDA 11.3) using Python 3.7 and PyTorch 1.11. Models were trained on KITTI odometry and evaluated on SemanticKITTI. Training utilized AdamW with a learning rate of 1 × 10^−5^ and a decay factor of γ = 0.95. Images of resolution 1220 × 370 were processed with a batch size of 4. The probabilistic ray sampler employed k = 3 Gaussians and m = 4 samples per Gaussian. Full experiments ran for 50 epochs, with ablation runs limited to 20 epochs.

### 4.3. Experimental Results

To assess the generalization capability of our method, all experiments were conducted using Sequence 08, which was deliberately withheld during training to ensure an unbiased evaluation. The evaluation focused on the model’s ability to infer depth from a single input image, estimating depth for any frame located within a 10 m radius of the input view. The corresponding experiment results are shown in Table 1.

To assess the model’s performance, the 0–10 m depth range was divided into 1 m intervals. For each interval, 585 reference images were selected, yielding a total of 9349 monocular inputs. Quantitative results show a gradual degradation in accuracy as the distance from the input frame increases, while the proposed method still maintains strong performance across all metrics.

As shown in Table 1, the model demonstrates impressive generalization ability, effectively adapting to previously unseen scenes. Moreover, compared to traditional monocular depth estimation techniques, this method is capable of synthesizing novel depth information beyond the original viewpoint, offering substantial value for real-world deployment in autonomous driving applications.

For further evaluating both effectiveness and computational efficiency, this study compares the proposed approach with other NeRF-based monocular depth estimation methods shown in Table 2, where Frames Per Second (FPS) and GPU Memory Usage are reported as efficiency indicators.

Table 2 shows the proposed approach surpasses all baseline models in NeRF-based monocular depth estimation with less computational cost, delivering notable gains on the more challenging evaluation metrics. For instance, in terms of Abs Rel and δ_1_, the method yields evaluation results of 0.1710 and 74.77, respectively, whereas VisionNerf—the top-performing baseline—achieves 0.2054 and 69.11, which demonstrates a clear advantage. Although the gap in RMSE is relatively small, it is noteworthy that this method excels in close-range depth estimation, which is identical to the results in Table 1.

Existing NeRF-based depth estimation approaches like PixelNeRF shares the closest design philosophy with the proposed approach, as both operate using single-image inputs. However, as indicated in Table 2, our method demonstrates superior performance compared to PixelNeRF, achieving significantly improved results, this highlights its greater suitability for large-scale and complex environments encountered in autonomous driving.

Moreover, this study also investigates the auxiliary task of novel view synthesis. The corresponding results presented in Table 3. Notably, despite relying entirely on self-supervised training, the proposed framework demonstrates competitive performance in this task. Consistent with the trend observed in depth estimation, the quality of novel view synthesis degrades progressively as the distance from the input viewpoint increases.

A qualitative view-synthesis comparison was also conducted against the three methods summarized in Table 4. The proposed approach matches state-of-the-art performance and exceeds PixelNeRF on all evaluation metrics. This demonstrates that, despite relying solely on self-supervised training, the method retains robust view-synthesis effectiveness.

**Figure 5:**
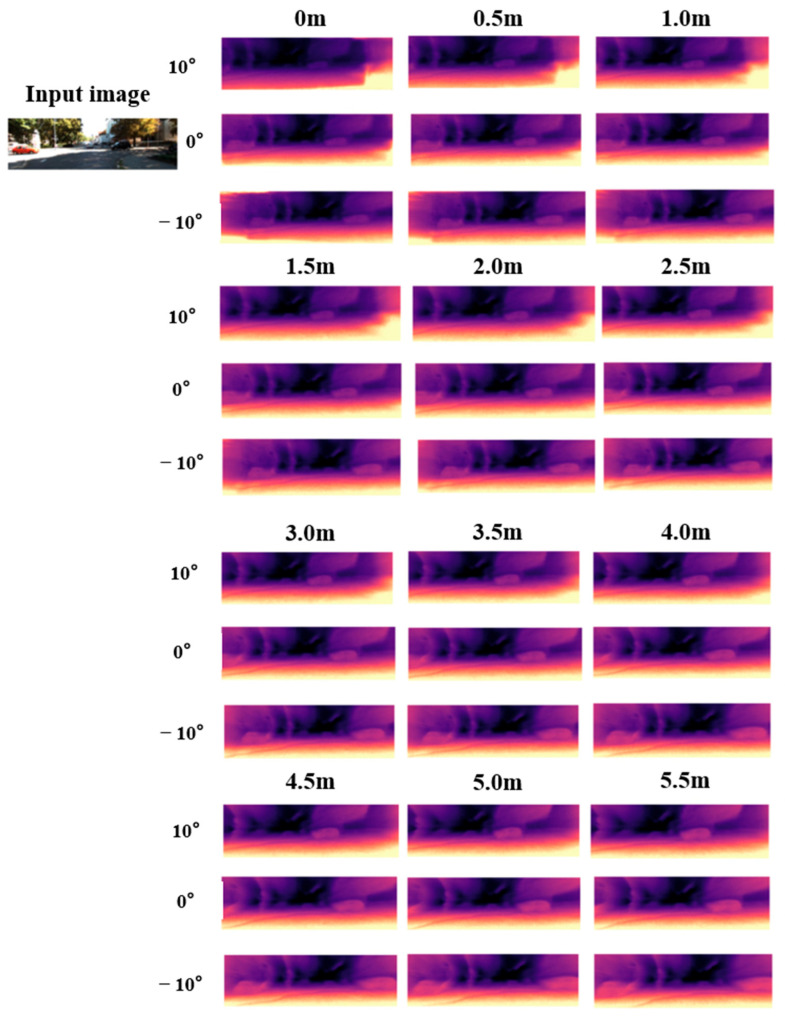
Depth estimation visualization results (0–5.5 m).

**Figure 6:**
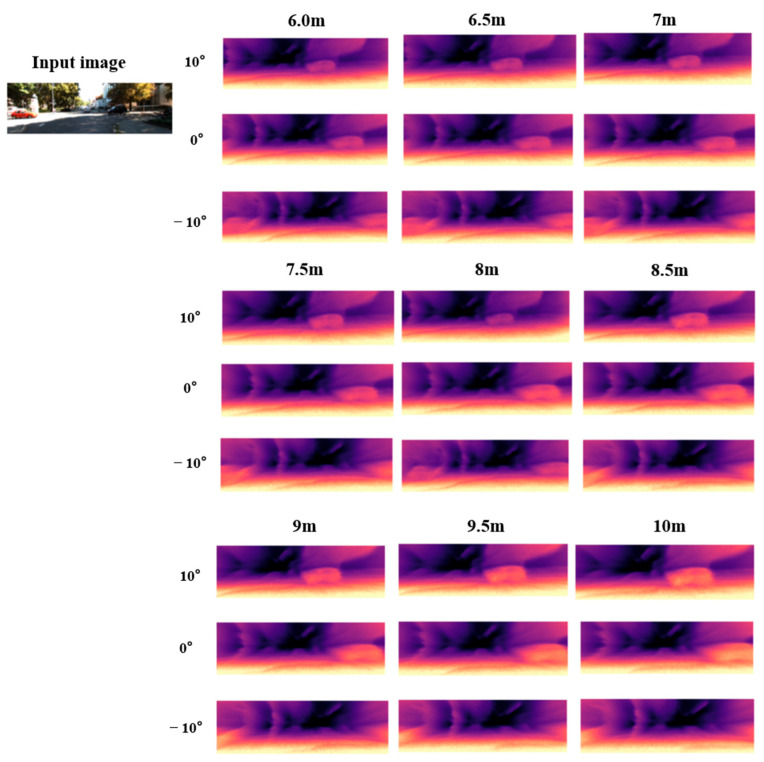
Depth estimation visualization results (6–10.0 m).

In addition to the quantitative analysis, we also generate new depth maps at different positions and viewpoints through single-image inference, as illustrated in Figure 5 and Figure 6.

Figure 5 and Figure 6 compare the method proposed in this chapter with traditional self-supervised monocular depth estimation approaches. The results show that NeRF-based monocular depth estimation effectively mitigates the scale ambiguity and artifact issues inherent to conventional methods while extending the valid depth estimation range. With only a single input image, the proposed method can infer depth within a 0–10 m range and expand the effective viewing angle by approximately 20°. As illustrated in the figures, our approach provides substantially clearer depth predictions at longer distances.

**Figure 7:**
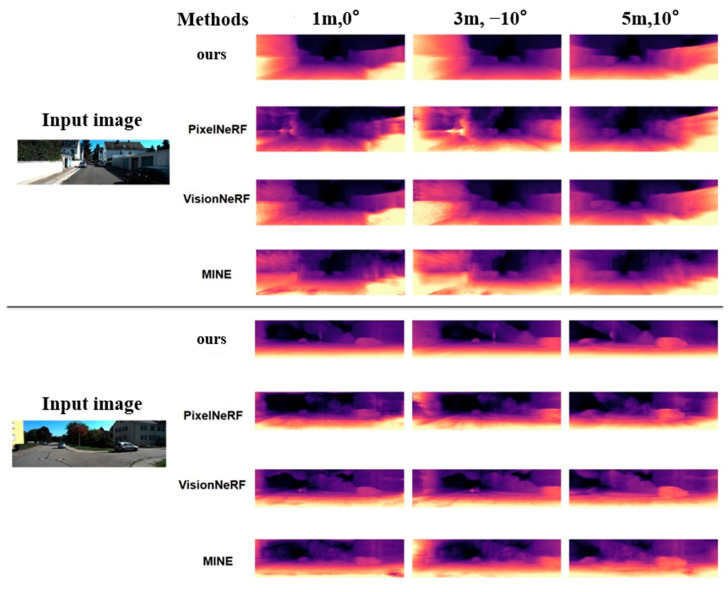
Visual Comparison Between the Proposed Method and Other Approaches.

In addition, Figure 7 presents a qualitative comparison between the proposed method and existing NeRF-based monocular depth estimation approaches. The synthesized depth results from three distinct viewpoints demonstrate that our method yields higher visual fidelity, particularly in maintaining sharper and more accurate depth boundaries. Notably, the depth predictions for the original input view remain stable under ±10° viewpoint variations. Compared with alternative methods, our approach produces more consistent depth maps with significantly fewer edge artifacts, indicating improved robustness across diverse viewing perspectives.

To further investigate the contributions of each component in the algorithm, ablation experiments were conducted on the probabilistic sampling module, spherical network with adaptive fine-grained channel attention mechanism, and loss functions (standard L1 reconstruction loss $L_{rgb}$ and reprojection loss $L_{reproj}$.

For the spherical network with adaptive fine-grained channel attention, a standard U-Net with similar capacity was used as a replacement in ablation experiments. The probabilistic sampling module was substituted with the standard hierarchical sampling used in NeRF. The specific evaluation results are shown in Table 5.

The table shows all proposed modules contribute to the optimal depth estimation performance for autonomous driving vehicles. Notably, the application of the reprojection loss $L_{reproj}$ leads to a significant improvement in the Abs Rel metric, demonstrating its beneficial effect on close-range depth estimation.

To further illustrate the benefits of the proposed reprojection loss $L_{reproj}$, this experiment applies $L_{reproj}$ to other comparative methods. Results are shown in Table 6. As indicated by the table, all baseline methods demonstrate performance improvements when augmented with reprojection loss.

In addition to ablation studies on the core model components and loss functions, this work also explores the effects of varying the number of Gaussian functions in the probabilistic ray sampling module, as well as the number of sampling points assigned to each Gaussian. The corresponding experimental results are shown in Table 7.

Our analysis reveals that excessive Gaussian functions (k) or sampling points (m) do not necessarily enhance density approximation quality. The optimal configuration happens at k=4 and m=8, yielding superior accuracy without unnecessary complexity.

The above experiments comprehensively evaluate the proposed method and demonstrate its superior performance compared with existing approaches, particularly in terms of sampling efficiency and long-range depth estimation accuracy. But it should be noted that although the KITTI dataset is widely adopted, it does not fully represent all complex real-world conditions. Future work will further validate the proposed method on additional datasets.

## 5. Conclusions and Future Work

This paper presents GaussNeRF, a NeRF-based monocular depth estimation method for autonomous driving. The proposed method introduces two key innovations: (1) A fine-grained adaptive channel attention mechanism embedded in a spherical network architecture, which enhances field-of-view coverage and feature extraction precision. (2) A Gaussian probabilistic ray sampling strategy that improves computational efficiency in large-scale scenes. By projecting 2D image features onto 3D sampling points, the proposed framework achieves superior performance. Comprehensive evaluations on the KITTI benchmark demonstrate that our approach outperforms existing state-of-the-art methods, particularly in occluded region reconstruction and large-scale environment processing. From a practical perspective, the reduced sampling density and computational overhead make the proposed method well suited for real-world autonomous driving systems, where efficiency, scalability and cost-effectiveness are critical requirements.

Future work will focus on accelerating inference, enhancing adaptation to dynamic environments, improving robustness under diverse weather conditions, exploring efficient sensor fusion strategies and validating the proposed method on additional datasets. These advances strengthen the applicability of monocular depth estimation in practical autonomous driving environments.

## Figures and Tables

**Table 1:** Depth Evaluation Results at Different Distances. The arrows (↑, ↓) indicate an increase and a decrease, respectively.

Distance	Abs Rel ↓	Sq Rel ↓	RMSE ↓	RMSE Log ↓	δ1 ↑	δ2 ↑	δ3 ↑	Input Frame
1	0.1433	0.976	4.974	0.2332	0.810	0.930	0.968	585
2	0.1494	1.120	5.385	0.2489	0.797	0.920	0.962	970
3	0.1534	1.139	5.394	0.2555	0.788	0.915	0.959	980
4	0.1590	1.212	5.606	0.2660	0.775	0.906	0.954	985
5	0.1654	1.247	5.639	0.2754	0.760	0.899	0.951	976
6	0.1724	1.337	5.861	0.2881	0.744	0.888	0.944	977
7	0.1784	1.380	5.939	0.2973	0.731	0.881	0.940	964
8	0.1858	1.480	6.171	0.3097	0.714	0.870	0.934	980
9	0.1932	1.581	6.385	0.3231	0.697	0.859	0.927	982
10	0.1992	1.607	6.418	0.3324	0.684	0.851	0.922	950
Sum	0.1710	1.321	5.810	0.2849	0.748	0.890	0.945	9349

**Table 2:** Comparison with Other NeRF-based Monocular Depth Estimation Methods. The arrows (↑, ↓) indicate an increase and a decrease, respectively.

Methods	Memory ↓	FPS ↑	Abs Rel ↓	Sq Rel ↓	RMSE ↓	RMSE Log ↓	δ1 ↑	δ2 ↑	δ3 ↑
PixelNeRF [44]	1384 MB	5.6	0.2364	2.080	6.449	0.3354	0.658	0.854	0.929
MINE [45]	1275 MB	6.5	0.2248	1.787	6.343	0.3283	0.659	0.855	0.933
VisionNerf [46]	1016 MB	7.1	0.2054	1.490	5.841	0.3073	0.691	0.883	0.944
Ours	615 MB	14.8	0.1710	1.321	5.810	0.2849	0.748	0.890	0.955

**Table 3:** View synthesis results at different distances. The arrows (↑, ↓) indicate an increase and a decrease, respectively.

Distance	PSNR ↑	SSIM ↑	LPIPS ↓	Input Frame
1	19.49	0.644	0.312	588
2	18.57	0.600	0.359	964
3	17.78	0.555	0.410	979
4	17.10	0.517	0.449	979
5	16.47	0.484	0.483	983
6	16.20	0.458	0.509	971
7	15.64	0.437	0.531	968
8	15.21	0.414	0.550	982
9	14.76	0.392	0.569	980
10	14.43	0.375	0.583	945
Sum	16.43	0.482	0.482	9349

**Table 4:** Comparison of view synthesis results between the proposed method and other approaches. The arrows (↑, ↓) indicate an increase and a decrease, respectively.

Method	PSNR ↑	SSIM ↑	LPIPS ↓
PixelNeRF	15.80	0.466	0.489
MINE	16.03	0.496	0.448
VisionNerf	16.49	0.483	0.468
Ours	16.43	0.482	0.482

**Table 5:** Ablation Evaluation Results of Modules and Loss Functions. The arrows (↑, ↓) indicate an increase and a decrease, respectively.

Method	Abs Rel ↓	Sq Rel ↓	RMSE ↓	RMSE Log ↓	δ1 ↑	δ2 ↑	δ3 ↑
Ours	0.1717	1.309	5.696	0.2809	75.01	89.35	94.76
$\mathrm{No}\;L_{rgb}$	0.1911	1.639	6.826	0.3730	69.76	85.99	92.78
$\mathrm{No}\;L_{reproj}$	0.1926	1.471	5.890	0.2949	71.82	88.64	94.49
No sphere U-Net	0.1766	1.379	5.897	0.2943	73.78	88.26	94.08
No-Sample	0.1845	1.318	5.763	0.2880	71.60	89.25	94.71

**Table 6:** Ablation Experiments on Reprojection Loss. The arrows (↑, ↓) indicate an increase and a decrease, respectively.

Methods	$L_{reproj}$	Abs Rel ↓	Sq Rel ↓	RMSE ↓	RMSE Log ↓	δ1 ↑	δ2 ↑	δ3 ↑
PixelNeRF		0.2364	2.080	6.449	0.3354	65.81	85.43	92.90
	√	0.1986	1.544	5.963	0.3093	70.30	87.19	93.82
MINE		0.2248	1.787	6.343	0.3282	65.87	85.52	93.30
	√	0.2003	1.599	6.023	0.3070	70.22	86.98	93.89
VisionNerf		0.2054	1.490	5.841	0.3073	69.11	88.28	94.37
	√	0.1749	1.380	5.643	0.2841	75.77	89.25	94.58

**Table 7:** Ablation study results of the probabilistic ray sampling module. The arrows (↑, ↓) indicate an increase and a decrease, respectively.

k	m	Abs Rel ↓	Sq Rel ↓	RMSE ↓	RMSE log ↓	δ1 ↑	δ2 ↑	δ3 ↑
1	32	0.1850	1.358	5.956	0.2940	71.38	88.73	94.51
2	16	0.1788	1.327	5.889	0.2878	72.68	88.90	94.70
4	4	0.1845	1.371	5.878	0.2940	71.62	88.59	94.51
	8	0.1717	1.309	5.696	0.2809	75.01	89.35	94.76
	16	0.1664	1.319	5.980	0.2894	74.58	88.48	94.71
8	2	0.1832	1.333	5.863	0.2934	71.60	88.61	94.50
	4	0.1768	1.311	5.824	0.2910	72.86	88.60	94.42
	8	0.1697	1.311	5.794	0.2873	74.59	88.71	94.34

## Data Availability

The data presented in this paper can be acquired from https://www.cvlibs.net/datasets/kitti/ (accessed on 12 June 2024).

## Abbreviations

The following abbreviations are used in this manuscript:

NeRF	Neural Radiance Field
SE	Squeeze-and-Excitation
Abs Rel	Absolute Relative Error
RMSE	Root Mean Square Error
PSNR	Peak Signal-to-Noise Ratio
MLP	Multi-Layer Perceptron
GMM	Gaussian Mixture Model
FCA	Fine-Grained Channel Attention
VGG	Visual Geometry Group
CRF	Conditional Random Field
KL	Kullback–Leibler
PSOM	Probabilistic Self-Organizing Map
